# Elucidation of Desensitization Mechanisms Induced by Oral Immunotherapy in a Rat Model of Ovalbumin Allergy

**DOI:** 10.3390/foods14081424

**Published:** 2025-04-21

**Authors:** Daigo Takizawa, Tomoharu Yokooji, Chika Miyamoto, Yuki Koga, Keisuke Oda, Ryohei Ogino, Takanori Taogoshi, Hiroaki Matsuo

**Affiliations:** 1 Department of Pharmaceutical Services, Graduate School of Biomedical and Health Sciences, Hiroshima University, 1-2-3 Kasumi, Minami-ku, Hiroshima 734-8551, Japan; tdaigo@hiroshima-u.ac.jp (D.T.); b190027@hiroshima-u.ac.jp (C.M.); ykoga@hiroshima-u.ac.jp (Y.K.); taogo@hiroshima-u.ac.jp (T.T.); hmatsuo@hiroshima-u.ac.jp (H.M.); 2 Department of Pharmaceutical Services, Hiroshima University Hospital, 1-2-3 Kasumi, Minami-ku, Hiroshima 734-8551, Japan; 3 Department of Frontier Science for Pharmacotherapy, Graduate School of Biomedical and Health Sciences, Hiroshima University, 1-2-3 Kasumi, Minami-ku, Hiroshima 734-8553, Japan; ryogino@hiroshima-u.ac.jp; 4 Laboratory of Biopharmaceutics and Pharmacokinetics, Faculty of Pharmaceutical Sciences, Hiroshima International University, 5-1-1 Hirokoshingai, Kure, Hiroshima 737-0112, Japan; oda@hirokoku-u.ac.jp

**Keywords:** food allergy, ovalbumin, oral immunotherapy, allergen-specific IgE, allergen-specific IgG

## Abstract

Oral immunotherapy (OIT) is a promising approach for treating food allergy. Here, we elucidated the mechanisms of desensitization induced by OIT in rats sensitized to ovalbumin (OVA). The desensitization was induced by ingestion of OVA three times per week after sensitization in rats. OIT suppressed the decrease in rectal temperature and increase in plasma histamine levels induced by OVA injection immediately and 4 weeks after OIT completion. Plasma OVA-specific IgE (sIgE) levels did not differ between the non-OIT and OIT groups, but OVA-specific IgG_1_ levels were higher in the OIT group than in the non-OIT group at both timepoints. To evaluate IgG’s effect on IgE crosslinking with OVA, amplified luminescence proximity homogeneous assay involving crosslinking (AlphaCL) was performed. When IgG was removed using a Protein G column, the AlphaCL signal was significantly increased, especially in the OIT group, indicating that OIT-induced IgG inhibited the sIgE response. The proportions of cluster of differentiation (CD)4^+^ cells and CD4^+^CD25^+^FoxP3^+^ cells in mesenteric lymph nodes and spleen were similar between the two groups. These findings indicate that OIT attenuates systemic allergic responses by inhibiting sIgE binding to OVA through increased IgG. Our model is useful for understanding the mechanisms of OIT and optimizing therapeutic strategies for ameliorating food allergies.

## 1. Introduction

The prevalence of food allergies has been increasing worldwide [1,2]. In the Japanese population, 5–10% of infants, 5% of young children, and 4.5% of school-aged children suffer from food allergies [3]. Food allergies can be triggered by ingestion of various foods, including hen eggs, cow milk, tree nuts, wheat, and peanuts. Among these foods, hen eggs are the most common food allergen in Japan. Most patients with food allergy develop immediate-type allergic symptoms, which are caused by crosslinking of multiple immunoglobulin (Ig)E antibodies specific for a food allergen (sIgE) that bind to high-affinity IgE receptors on mast cells in complex with the ingested allergen. Patients with food allergy can suffer from skin manifestations, gastrointestinal and respiratory symptoms, and even fatal anaphylactic shock [3]. Patients should strictly avoid consuming trigger foods to prevent the emergence of symptoms because no curative therapy for food allergy has been established. However, the strict avoidance of food can place a significant burden on patients and their families. Patients are also at risk of anaphylaxis due to the accidental ingestion of food allergens. It has also been reported that patients strictly avoiding a particular food had a lower threshold for developing allergic symptoms and lower tolerance rates to it than those not performing such food avoidance [4].

Oral immunotherapy (OIT), in which regular ingestion of a causative food induces immunotolerance to a causative allergen, has been highlighted as a promising curative therapy for food allergies. Several clinical studies have reported that OIT was effective in preventing the elicitation of IgE-mediated allergic symptoms to various foods [5,6]. For example, Miyaji et al. reported that ultra-low-dose OIT could induce immunotolerance and increase the tolerable dose in oral food challenge without anaphylaxis in pediatric patients with immediate-type allergy to hen eggs or cow milk [7]. Meanwhile, Vickery et al. reported that 12 of 24 patients with peanut allergy who received OIT did not show any allergic symptoms during a provocation test 4 weeks after OIT was discontinued [8]. By contrast, various cases in which OIT showed insufficient efficacy for certain foods have been reported. For example, Furuta et al. reported that exercise-induced allergic reactions on desensitization remained for a long period after OIT in patients with wheat allergy [9]. OIT can cause anaphylaxis in patients with food allergy because the patient ingests food containing the causative allergen. Therefore, it is detrimental to the patient to perform ineffective OIT on a patient. The mechanisms of OIT-induced desensitization should be elucidated to establish efficient and safe OIT. Although the detailed mechanism of OIT is not fully understood, decreased plasma sIgE levels [8], decreased basophil response [10], increased serum sIgG_4_ levels [11], and induction of regulatory T cells (Treg) [12] have been observed in patients with food allergy successfully desensitized with OIT.

Several animal models have been established to elucidate the mechanisms of OIT-induced desensitization. However, most of these studies evaluated the prophylactic effect against allergen sensitization with an allergen by the induction of oral tolerance (OT) before sensitization [13,14,15]. Conversely, few models have been established to evaluate the efficacy of desensitization to causative allergens by inducing OT after sensitization. Furthermore, the efficacy of OIT differed among each OIT model. For example, Leonard et al. established a model for the induction of OIT by administering gradually increased doses of egg white to mice orally sensitized with ovalbumin (OVA) [16]. In their OIT mouse model, oral OVA challenge did not decrease body temperature on the first day after OIT, despite higher serum levels of OVA-sIgE than in non-OIT mice. Meanwhile, Tordesillas et al. attempted to induce OIT in mice orally sensitized with OVA by regularly administering a consistent dose of OVA, but the degree of body temperature reduction due to OVA challenge and serum levels of OVA-sIgE did not differ between the non-OIT and OIT groups [17]. This background highlights the need for further studies to elucidate the mechanisms of OIT in an OIT animal model. Mice have often been used to establish models of food allergy and OT. However, using mouse models for analyses that require a large volume of serum samples, such as measuring antibody levels over time or multiple antibody levels in a single individual, has been difficult. In this study, we aimed to establish a rat model of OIT. Knippels et al. reported that the serum OVA-sIgE level in Brown Norway (BN) rats was the highest among four strains (BN, Hooded Lister, Wistar, and Piebald Virol Glaxo rats) intraperitoneally sensitized with OVA [18]. Meanwhile, Pilegaard and Madsen also reported that female BN rats had significantly higher OVA-sIgE levels and numbers of responders than male rats when both sexes were given oral OVA daily for 35 days [19]. In this context, we aimed to establish an OIT model using female BN rats and to elucidate the mechanisms of OIT-induced desensitization.

## 2. Materials and Methods

### 2.1. Animals

Female BN rats (3 weeks old) purchased from Japan SLC (Hamamatsu, Japan) were fed a standard laboratory diet (MF; Oriental Yeast, Tokyo, Japan) and water ad libitum for 1 week before sensitization with OVA (Sigma-Aldrich, St. Louis, MO, USA). The number of rats per group was determined based on those reported previously [20,21]. All animal experiments were conducted in accordance with the Guide for Animal Experimentation of the Committee of Research Facilities for Laboratory Animal Sciences of Hiroshima University (Approval No. A21-148-2; Approval date, 10 February 2022; Hiroshima, Japan).

### 2.2. Sensitization and OIT Protocol

**Figure 1:**
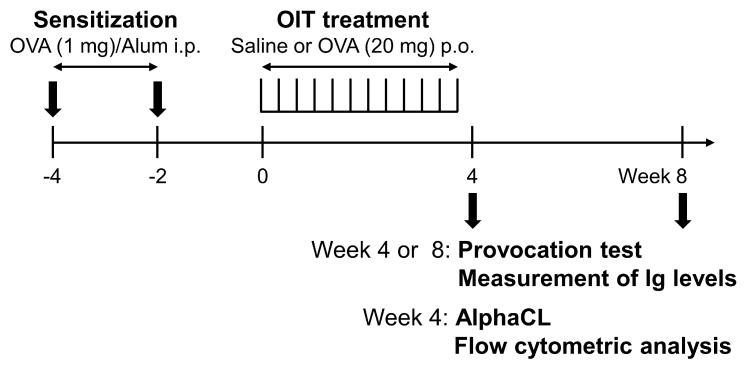
Sensitization and oral immunotherapy (OIT) to OVA (schematic). Brown Norway rats were administered OVA intraperitoneally at 2 and 4 weeks before the initiation of OIT for sensitization. At 2 weeks after the final immunization (week 0), rats were orally administered vehicle alone (saline) or 20 mg of OVA three times a week for a period of 4 weeks for OIT. Blood was collected from the jugular vein at weeks 0 and 4 (or 8) to determine the levels of OVA-sIgE and-sIgG subclasses in plasma. Provocation test with intravenous OVA injection was performed to measure rectal temperature and plasma histamine levels. At week 4, AlphaCL and flow cytometric analyses were performed. Ig, immunoglobulin; i.p., intraperitoneal administration; p.o., per os (oral administration).

Rats were sensitized with OVA, as shown in Figure 1, in accordance with our previously reported method [20]. Briefly, OVA was dissolved in a mixture of physiological saline and 2% Alhydrogel^®^ adjuvant (InvivoGen, San Diego, CA, USA) (volume ratio, 2:1) to a concentration of 2 mg/mL. The prepared solution (0.5 mL) was intraperitoneally injected into rats at 2 and 4 weeks before OIT initiation for sensitization. Two weeks after the final sensitization (week 0), the rats were divided into two groups, a non-OIT group and an OIT group, to ensure equal plasma OVA-sIgE levels (optical density: non-OIT group, 0.48 ± 0.08 absorbance unit [AU] vs. OIT group, 0.40 ± 0.07 AU; *p* = 0.390). These rats were orally administered 0.5 mL of physiological saline alone (non-OIT group) or physiological saline containing 20 mg of OVA (OIT group) three times a week for a period of 4 weeks using a stainless-steel gavage tube. Blood was collected from the jugular vein at weeks 0 and 4 (or 8) to determine the levels of OVA-sIgE and sIgG subclasses in plasma.

### 2.3. Measurement of Plasma Levels of OVA-sIgE and sIgG Subclasses

ELISA was conducted to determine the plasma levels of OVA-sIgE using a slightly modified version of our previously reported method [14]. Briefly, 100 ng of MARE-1 (GeneTex, Irvine, CA, USA) was coated onto each well of a plate (Thermo Fisher Scientific, Waltham, MA, USA) overnight at 4 °C. The wells were washed with phosphate-buffered saline (PBS; pH 7.4) containing 0.1% Tween 20 (PBS-T) and blocked with 1% Block Ace^®^ (DS Pharma Biomedical, Osaka, Japan) for 1 h at 37 °C. Plasma samples were diluted 1:10 with 1% Block Ace^®^ and were added to the wells. After incubation for 1 h at 37 °C, the wells were incubated with biotinylated OVA for 1 h at 37 °C. Then, each well was incubated with horseradish peroxidase (HRP)-conjugated streptavidin (Proteintech Japan, Tokyo, Japan) at 37 °C for 1 h. After removal of the HRP-conjugated streptavidin, each well was incubated with 3,3′,5,5′-tetramethylbenzidine (TMB; SeraCare Life Sciences, Gaithersburg, MD, USA) for 15 min at 37 °C. The reaction was terminated by the addition of 1 M phosphoric acid. Absorbance was measured at 450 nm, with that at a wavelength of 630 nm used as a reference, using a Multiskan GO spectrophotometer (Thermo Fisher Scientific).

To determine the levels of OVA-sIgG and its subclasses in plasma, each well was coated with OVA (100 ng) overnight at 4 °C. After washing with PBS-T, wells were blocked with 1% Block Ace^®^ for 2 h at 37 °C. Plasma was diluted as follows: 1:30,000 for total OVA-sIgG and sIgG_1_, 1:2000 for sIgG_2a_, and 1:50 for sIgG_2b_ and sIgG_2c_ in 1% Block Ace^®^. The diluted plasma was added to each well, followed by incubation at 37 °C for 2 h. The wells were then incubated with HRP-conjugated goat anti-rat IgG (H + L) (diluted 1:100,000 in PBS; Bethyl Laboratories, Montgomery, TX, USA), goat anti-rat IgG_1_ (diluted 1:100,000 in PBS; Bethyl Laboratories), goat anti-rat IgG_2a_ (diluted 1:30,000 in PBS; Bethyl Laboratories), goat anti-rat IgG_2b_ (diluted 1:30,000 in PBS; Invitrogen, Carlsbad, CA, USA), or goat anti-rat IgG_2c_ (H) (diluted 1:30,000 in PBS; Bethyl Laboratories) at 37 °C for 1 h. After washing, TMB was added to measure the absorbance, as in the sIgE assay.

### 2.4. Provocation Test

To evaluate systemic allergic reactions, changes in rectal temperature and plasma histamine levels were determined following intravenous OVA challenge, in accordance with a previous report [14]. Briefly, medetomidine (0.15 mg/kg), midazolam (2 mg/kg), and butorphanol (2.5 mg/kg) were injected intraperitoneally into the rats for anesthesia. A rectal thermometer (Shibaura Electronics, Saitama, Japan) was inserted into the rectum through the anus. After the rectal temperature had stabilized, OVA dissolved in physiological saline (10 mg/mL) was injected via the jugular vein at a dose of 1 mL/kg (0 min). Rectal temperature was monitored before (0 min) and 30 min after OVA injection. Furthermore, 0.25 mL of blood was obtained at 0, 1, 5, and 15 min via another jugular vein to measure the histamine levels in plasma. At the end of the experiment (week 4), whole blood was obtained through the abdominal aorta to detect OVA-sIgE in plasma using amplified luminescence proximity homogeneous assay involving crosslinking (AlphaCL), which we previously developed to detect allergen-sIgE with high-affinity IgE receptor alpha subunit (FcεRIα) crosslinking ability [22,23]. The blood was centrifuged, and the plasma was stored at −30 °C until use. The mesenteric lymph nodes (MLN) and spleen were also isolated to evaluate the proportions of cluster of differentiation (CD)4^+^ cells and CD4^+^CD25^+^FoxP3^+^ cells.

### 2.5. Determination of Plasma Histamine Levels

The plasma samples were deproteinized with acetonitrile, in which the final concentration of acetonitrile was 80%. The samples were centrifuged at 3000× *g* for 10 min, and the obtained supernatant was used for analysis. Plasma concentrations of histamine were determined using liquid chromatography–tandem mass spectrometry (LCMS-8040 triple quadrupole mass spectrometer; Shimadzu, Kyoto, Japan) with a YMC-Triart C18 column (YMC, Kyoto, Japan). The column was kept at 40 °C, and the temperature of the sample cooler was 4 °C. The mobile phase consisted of solvent A (10 mM ammonium formate, pH 4.0) and solvent B (acetonitrile). The elution of histamine was performed using the following linear gradient: 20% of B for 3 min, 20% to 70% of B for 3–5 min, 70% of B for 5–6 min, and 20% of B for 6–9 min with a flow rate of 0.5 mL/min. Ionization was conducted in positive electrospray ionization (ESI) mode at 250 °C, with transitions of *m*/*z* = 112.10 and *m*/*z* = 95.10 monitored for precursor ion and product ions, respectively. The calibration curve for histamine showed a linear response across a concentration range of 0.10–3000 ng/mL.

### 2.6. Detection of OVA-sIgE in Plasma Using AlphaCL

Plasma OVA-sIgE levels were detected using AlphaCL, in accordance with our previous report [22]. OVA-sIgE was detected using AlphaCL after IgG removal through a Protein G column (Cytiva, Tokyo, Japan), which efficiently depleted IgG while preserving IgE [24]. Plasma from non-OIT and OIT rats collected immediately after OIT completion was diluted 1:1 in buffer (PerkinElmer, Waltham, MA, USA). These samples were divided into untreated and IgG-depleted groups. Plasma from the IgG-depleted group was treated in columns, in accordance with the manufacturer’s protocol. Each well was incubated with OVA (20 ng) and each plasma sample at room temperature for 1 h. FcεRIα protein-conjugated acceptor (1 µg) and donor beads (0.25 µg, PerkinElmer) suspended in 10 µL of buffer were added. Wells were incubated at room temperature for 24 h in the dark. Signals were determined using NIVO™ (PerkinElmer). The counts in buffer alone were subtracted from the total counts to calculate the net counts for each sample.

### 2.7. Flow Cytometric Analysis

Cells in MLN and spleen were dispersed in PBS containing 1% bovine serum albumin (BSA) and passed through a 70-µm cell strainer. The erythrocytes in the spleen were then lysed by adding RBC lysis buffer (pluriSelect Life Science, Leipzig, Germany) and centrifuged at 300× *g* for 10 min. The supernatant was removed, and the cells were resuspended in PBS containing 1% BSA. These cells were immunostained with CD4-fluorescein mouse IgG_2A_, CD25/IL-2 Rα-PE mouse IgG_1_, and FoxP3 Alexa Fluor^®^ 647 mouse IgG_1_ using the FlowX^TM^ Rat Regulatory T Cell Multi-Color Flow Cytometry Kit, in accordance with the manufacturer’s protocol (R&D Systems, Minneapolis, MN, USA). The proportions of CD4^+^ cells and CD4^+^CD25^+^FoxP3^+^ cells were analyzed using Guava^®^ easyCyte^TM^ 8 (Cytek Biosciences, Fremont, CA, USA).

### 2.8. Statistical Analysis

Data are shown as the mean ± standard error of the mean (SE). Differences in the mean values between groups were assessed using ANOVA, followed by Student’s *t*-test or paired *t*-test. A *p*-value of <0.05 was considered statistically significant. Statistical analysis was conducted using Statcel4 software (version 1.0; OMS Publishing Inc., Saitama, Japan).

## 3. Results

### 3.1. Effect of OIT on Systemic Allergic Reactions in Rats Sensitized with OVA

**Figure 2:**
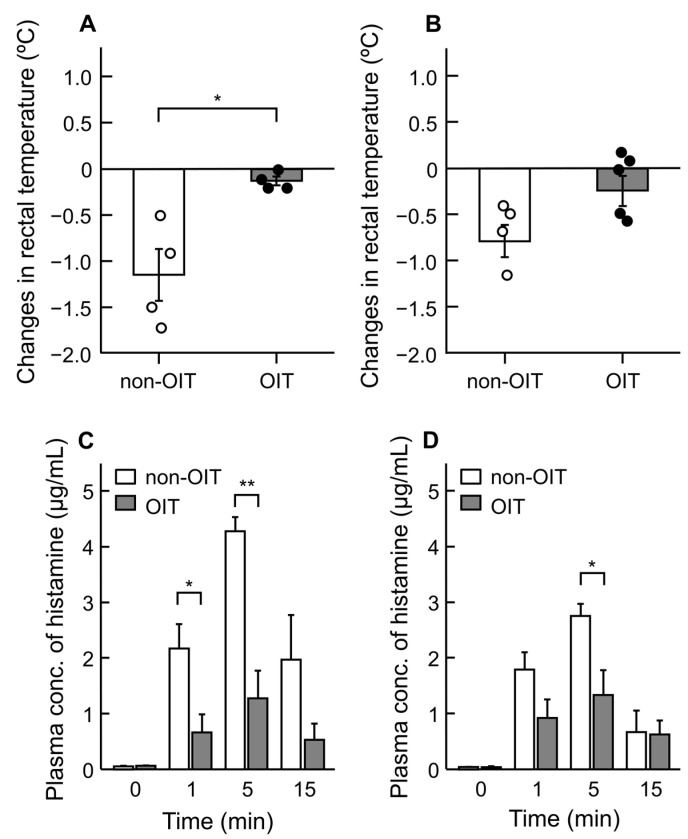
Effects of OIT on the systemic allergic reactions induced by provocation test in OVA-sensitized rats. Changes in rectal temperature (**A**,**B**) and plasma histamine levels before (0 min) and 1, 5, and 15 min after administration (**C**,**D**) were evaluated after the administration of OVA (10 mg) at week 4 (**A**,**C**) or week 8 (**B**,**D**). Each value represents the mean ± SE of four to five rats (non-OIT, *n* = 4; OIT, *n* = 4 at week 4, and non-OIT, *n* = 4; OIT, *n* = 5 at week 8). * *p* < 0.05 and ** *p* < 0.01: significantly different from the non-OIT group.

To evaluate the effects of OIT on systemic allergic reactions in rats sensitized with OVA, OVA was intravenously injected immediately (week 4) or 4 weeks after completion of OIT (week 8), followed by measurements of rectal temperature and plasma histamine levels (Figure 2). At week 4, the rectal temperature in the non-OIT group decreased by 1.2 °C within 30 min after intravenous OVA injection, whereas in the OIT group, this decrease was only 0.13 °C (*p* < 0.05) (Figure 2A). At week 8, the rectal temperature in the non-OIT group also decreased by 0.80 °C within 30 min after intravenous OVA injection, whereas that in the OIT group only decreased by 0.26 °C (Figure 2B).

Plasma histamine levels increased in the non-OIT and OIT groups 1 min after OVA injections, peaking at 5 min (Figure 2). At week 4, plasma levels of histamine were significantly lower in the OIT group (1.27 ± 0.50 µg/mL) than in the non-OIT group (4.28 ± 0.25 µg/mL, *p* < 0.05) 5 min after intravenous OVA injection (Figure 2C). The increase in plasma histamine levels observed 5 min after the OVA injection in the non-OIT group (2.76 ± 0.23 µg/mL) was also reduced by OIT at week 8 (1.34 ± 0.44 µg/mL, *p* < 0.05) (Figure 2D).

### 3.2. Effects of OIT on Plasma Levels of OVA-sIgE and -sIgG Subclasses

**Figure 3:**
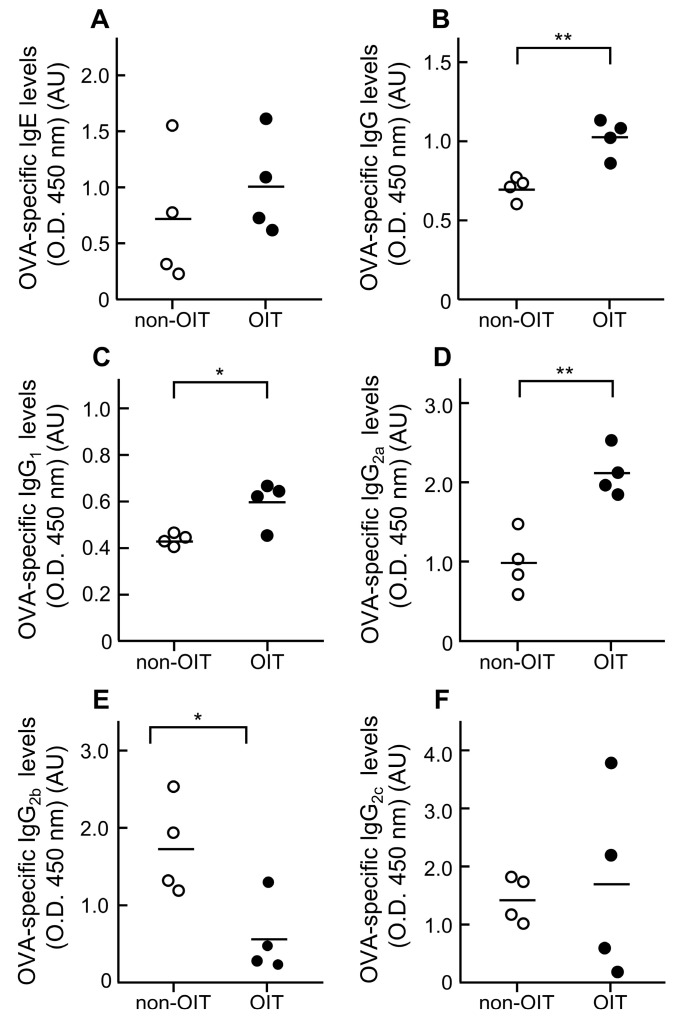
Effects of OIT on plasma levels of OVA-specific immunoglobulin in OVA-sensitized rats at week 4. Plasma levels of OVA-sIgE (**A**), sIgG (**B**), sIgG_1_ (**C**), sIgG_2a_ (**D**), sIgG_2b_ (**E**), and sIgG_2c_ (**F**) were determined using ELISA. These values represent the optical densities measured at 450 nm for plasma diluted 1:10 for sIgE, 1:30,000 for sIgG, 1:2000 for sIgG_2a_, and 1:50 for sIgG_2b_ and sIgG_2c_. Bars represent the mean values of four rats (non-OIT, *n* = 4; OIT, *n* = 4). * *p* < 0.05 and ** *p* < 0.01: significantly different from the non-OIT group.

**Figure 4:**
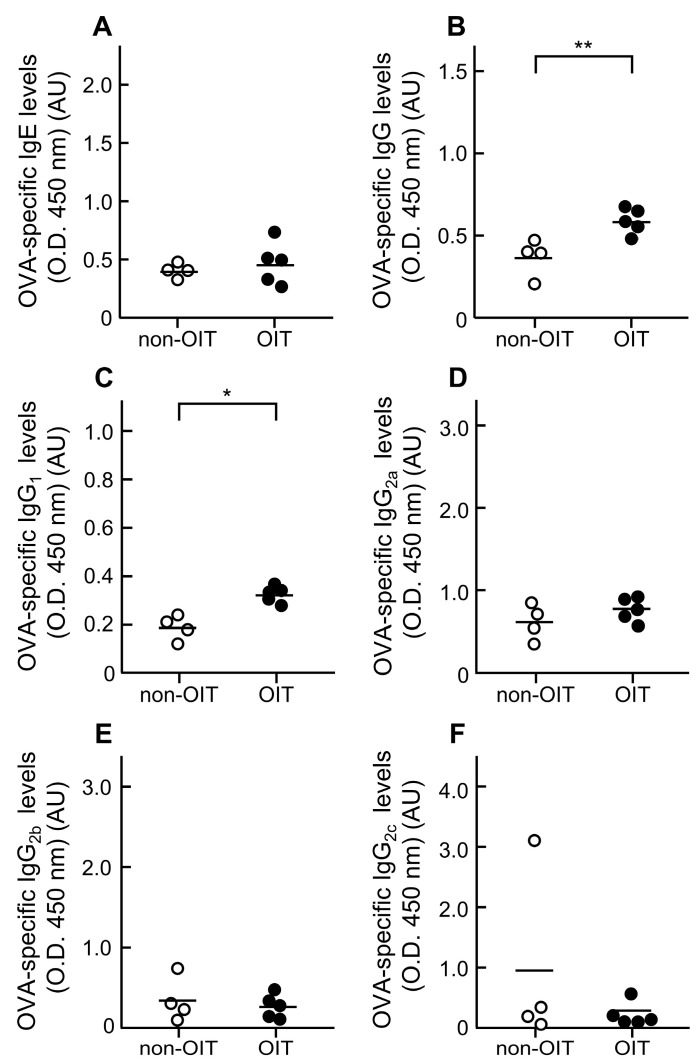
Effects of OIT on plasma levels of OVA-specific immunoglobulin in OVA-sensitized rats at week 8. Plasma levels of OVA-sIgE (**A**), sIgG (**B**), sIgG_1_ (**C**), sIgG_2a_ (**D**), sIgG_2b_ (**E**), and sIgG_2c_ (**F**) were determined using ELISA. These values represent the optical densities measured at 450 nm for plasma diluted 1:10 for sIgE, 1:30,000 for sIgG, 1:2000 for sIgG_2a_, and 1:50 for sIgG_2b_ and sIgG_2c_. Bars represent the mean values of four to five rats (non-OIT, *n* = 4; OIT, *n* = 5). * *p* < 0.05 and ** *p* < 0.01: significantly different from the non-OIT group.

To elucidate the mechanisms of desensitization induced by OIT, its effects on plasma levels of OVA-sIgE and -sIgG subclasses were determined using ELISA (Figure 3 and Figure 4). Regarding OVA-sIgE, no differences were observed between the OIT group and the non-OIT group at week 4 (non-OIT group, 0.72 ± 0.30 AU vs. OIT group, 1.00 ± 0.22 AU; *p* = 0.470) (Figure 3A) and week 8 (non-OIT group, 0.36 ± 0.03 AU vs. OIT group, 0.42 ± 0.07 AU; *p* = 0.564) (Figure 4A). By contrast, total OVA-sIgG levels were significantly higher in the OIT group than in the non-OIT group at week 4 (non-OIT group, 0.70 ± 0.04 AU vs. OIT group, 1.03 ± 0.06 AU; *p* < 0.01) (Figure 3B) and week 8 (non-OIT group, 0.36 ± 0.06 AU vs. OIT group, 0.58 ± 0.03 AU; *p* < 0.01) (Figure 4B). Among the OVA-sIgG subclasses, OVA-sIgG_1_ levels were also significantly higher in the OIT group than in the non-OIT group at week 4 (non-OIT group, 0.43 ± 0.01 AU vs. OIT group, 0.59 ± 0.05 AU; *p* < 0.05) (Figure 3C) and week 8 (non-OIT group, 0.18 ± 0.02 AU vs. OIT group, 0.31 ± 0.01 AU; *p* < 0.01) (Figure 4C). The OVA-sIgG_2a_ level was significantly higher in the OIT group (2.1 ± 0.15 AU) than in the non-OIT group (0.98 ± 0.19 AU) at week 4 (*p* < 0.01) (Figure 3D), but it showed no difference between these groups at week 8 (non-OIT group, 0.60 ± 0.10 AU vs. OIT group, 0.75 ± 0.06 AU; *p* = 0.21) (Figure 4D). The OVA-sIgG_2b_ levels were significantly lower in the OIT group (0.55 ± 0.25 AU) than in the non-OIT group (1.72 ± 0.31 AU) at week 4 (*p* < 0.05) (Figure 3E), but no differences were observed between the two groups at week 8 (non-OIT group, 0.34 ± 0.14 AU vs. OIT group, 0.26 ± 0.07 AU; *p* = 0.61) (Figure 4E). The OVA-sIgG_2c_ levels did not differ between the OIT and non-OIT groups at both week 4 (non-OIT group, 1.42 ± 0.20 AU vs. OIT group, 1.68 ± 0.81 AU; *p* = 0.76) (Figure 3F) and week 8 (non-OIT group, 0.95 ± 0.73 AU vs. OIT group, 0.24 ± 0.09 AU; *p* = 0.31) (Figure 4F).

### 3.3. Evaluation of the Effect of IgG on IgE Binding to OVA Using AlphaCL

**Figure 5:**
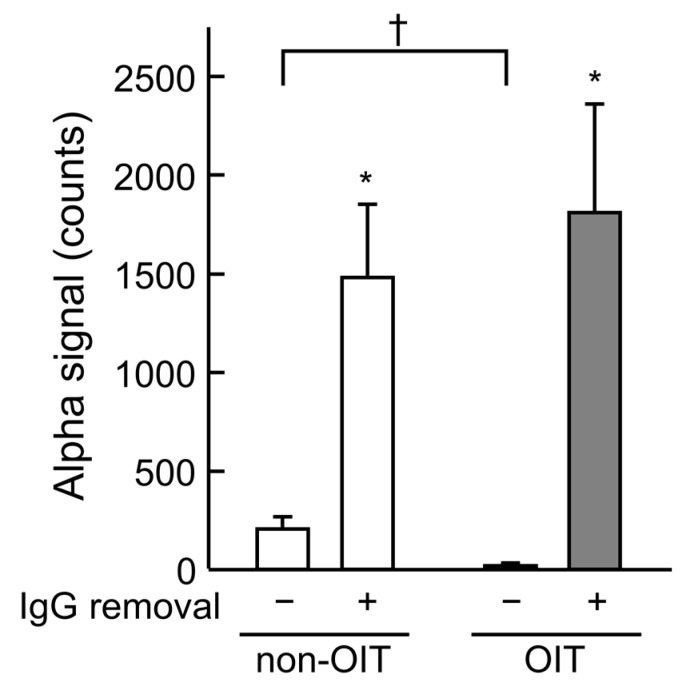
Effects of OIT and IgG removal from plasma on the detection of OVA-sIgE using AlphaCL. Plasma of non-OIT and OIT rats at 4 weeks was processed using the Protein G HP SpinTrap™ column. Net counts were calculated from the total counts in each sample by subtracting the counts in buffer without sIgE. Each value represents the mean ± SE of four rats (non-OIT, *n* = 4; OIT, *n* = 4). * *p* < 0.05: significantly different from the untreated plasma. ^†^ *p* < 0.05: significantly different from the non-OIT group.

The effect of plasma IgG on the IgE binding to OVA was evaluated using AlphaCL following the removal of IgG using Protein G columns (Figure 5). In untreated plasma, alpha signal was lower in the OIT group (22 ± 13 counts) than in the non-OIT group (206 ± 63 counts; *p* < 0.05). The signal was significantly increased by the removal of IgG in the non-OIT (untreated plasma, 206 ± 63 counts vs. IgG-depleted plasma, 1480 ± 372 counts; *p* < 0.05) and OIT groups (untreated plasma, 22 ± 13 counts vs. IgG-depleted plasma, 1812 ± 549 counts; *p* < 0.05).

### 3.4. Proportions of CD4^+^ Cells and CD4^+^CD25^+^FoxP3^+^ Cells in MLN and Spleen

**Figure 6:**
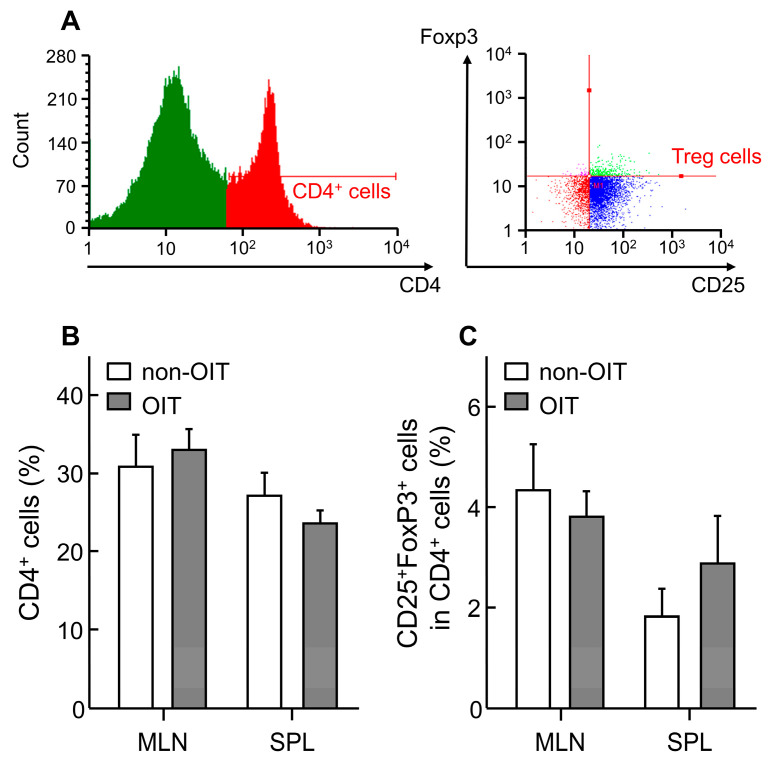
Effects of OIT on proportions of CD4^+^ cells and CD4^+^CD25^+^FoxP3^+^ cells in mesenteric lymph nodes (MLN) and spleen (SPL). Representative flow cytometric data for CD4^+^ cells and CD4^+^CD25^+^FoxP3^+^ cells (**A**). Populations of CD4^+^ cells relative to whole cells (**B**) and CD25^+^FoxP3^+^ cells relative to CD4^+^ cells (**C**). Cells were isolated from MLN and spleen of the non-OIT and OIT rats at 4 weeks. Each value represents the mean ± SE of four rats (non-OIT, *n* = 4; OIT, *n* = 4).

The proportions of CD4^+^ cells and Treg in the MLN and spleen were determined using flow cytometric analysis at week 4 (Figure 6A). The proportions of CD4^+^ cells in MLN (non-OIT, 30.8 ± 4.1% vs. OIT, 33.1 ± 3.4%; *p* = 0.68) and spleen (non-OIT, 27.3 ± 3.0% vs. OIT, 23.9 ± 1.67%; *p* = 0.35) did not differ between the non-OIT group and OIT group (Figure 6B). Meanwhile, the proportions of CD4^+^CD25^+^FoxP3^+^ cells in MLN (non-OIT, 4.34 ± 0.91% vs. OIT, 3.84 ± 0.50%; *p* = 0.64) and spleen (non-OIT, 1.86 ± 0.52% vs. OIT, 2.90 ± 0.93%; *p* = 0.37) were also similar between the two groups (Figure 6C).

## 4. Discussion

OIT has been highlighted as a promising curative treatment to induce desensitization in patients with food allergies. However, it remains unclear how OIT induces desensitization in patients who have already developed food allergy symptoms. Several animal models have been established to elucidate the mechanisms of OIT. However, most of them evaluate the prophylactic effect on sensitization to an allergen by the induction of OT before sensitization [13,14,15]. In this study, we established a rat model in which desensitization was induced by oral ingestion of OVA three times per week after sensitization. Using this model, we found that OIT increased the plasma levels of OVA-sIgG_1_, and that the increased IgG (presumably OVA-sIgG_1_) inhibited sIgE crosslinking with OVA, resulting in the suppression of degranulation and subsequent symptoms. Furthermore, desensitization induced by OIT persisted for at least 1 month after OIT treatment. To the best of our knowledge, this report is the first to show that OIT acts by increasing the inhibition of IgE-mediated degranulation by sIgG_1_ in an in vivo rat model.

The dose and frequency of allergen ingestion may affect the mechanism of OT induction. It was reported that anergy or T-cell depletion was induced by ingesting 500 mg of OVA five times in a mouse model, whereas these effects did not emerge with 0.5 mg [25]. In the present study, OVA-sensitized rats weighing approximately 130 g were given 20 mg of OVA orally three times a week for 4 weeks to induce desensitization. Nair and Jacob reported that the dose of compounds administered to rats should be divided by a factor (6.2) to convert it to a human equivalent dose [26]. Therefore, 20 mg/130 g body weight in rats is equivalent to 24.8 mg/kg body weight in humans. Assuming a childhood body weight of approximately 30–40 kg, this corresponds to the ingestion of approximately 740 to 990 mg of OVA in humans. Because egg white is composed of approximately 5.4% OVA [27], the dose of OVA administered to rats in our study is estimated to correspond to 13.7–18.3 g of egg white. In clinical studies of OIT, Fuentes-Aparicio et al. administered a maximum of 10 g of egg to children with egg allergy [28], while Burks et al. provided up to 10 g of egg white powder to children aged 5–18 years [29]. The doses of OVA in our study were slightly higher than those in these clinical studies, but our OIT rat model may reflect OIT in clinical settings.

When OVA was administered intravenously to rats immediately after OIT (week 4), rectal temperature decreased in the non-OIT group but decreased less in the OIT group (Figure 2A,B). In addition, plasma levels of histamine in the OIT group were lower than those in the non-OIT group (Figure 2C,D). Therefore, OIT effectively reduced the allergic responses after OVA challenge. The suppressive effect of OIT on allergic reactions persisted even 1 month after OIT, but the extent of this suppression tended to decrease. Therefore, the effect of desensitization induced by OIT in our rat model may be transient, which is consistent with some previous reports on human OIT [16,30,31].

An important finding of this study is the change in plasma levels of OVA-specific antibodies following OIT (Figure 3 and Figure 4). Plasma levels of OVA-sIgE did not differ between the non-OIT and OIT groups. These findings are consistent with those previously reported by Leonard et al. in a mouse model [16]. In humans, Vickery et al. reported that the change in serum peanut-specific IgE levels from baseline to the end of a peanut OIT study did not differ significantly between the placebo and OIT groups [32]. These findings suggest that the reduction in the development of allergic symptoms associated with mast cell and basophil degranulation by OIT is not due to the suppression of OVA-sIgE production. By contrast, OIT significantly increased the OVA-sIgG levels in plasma at week 4. The analysis of OVA-sIgG subclasses showed that OIT also increased the OVA-sIgG_1_ and -sIgG_2a_ levels in plasma but decreased the sIgG_2b_ levels. Furthermore, only increased OVA-sIgG_1_ levels were maintained in the OIT group at week 8, when desensitization was sustained. In AlphaCL, the signal counts in untreated plasma were lower in the OIT group than in the non-OIT group (Figure 5). This indicates that OIT suppresses the crosslinking of OVA-sIgE to OVA, which is consistent with the decreases in rectal temperature and histamine levels. Conversely, when IgG was removed from the plasma, signal counts increased in both the non-OIT and OIT groups compared with that for untreated plasma. Philips et al. reported that rat dinitrophenyl protein (DNP)-sIgG_1_ acted as a highly effective blocking antibody against the degranulation of rat basophil leukemia cells over a wide concentration range, whereas DNP-sIgG_2a_ could inhibit it only when immune complexes were formed with very low concentrations of high-valency DNP-human serum albumin [33]. In addition, the IgG levels in rat plasma are typically significantly higher than the IgE levels [34]. We speculate that the high plasma levels of OVA-sIgG, especially sIgG_1_, may have competitively inhibited IgE binding to allergens, thereby reducing the development of allergic symptoms. However, we cannot rule out the possibility that non-sIgG also inhibited IgE binding to OVA because IgG was removed simply using the Protein G column in this study. Further studies are necessary to determine the extent to which non-sIgG contributes to the inhibition of degranulation in OIT. It remains unclear whether the increase in sIgG_1_ observed in our rat model would also occur in humans because IgG subclasses differ between rats and humans. OIT has been shown to increase sIgG_4_ levels in patients with food allergy [11,32]. Therefore, our findings suggest that the OIT-induced increase in IgG subclasses may act as blocking antibodies to suppress IgE-mediated allergic reactions in humans.

Ingested food allergens can be taken up by a CD103^+^ regulatory dendritic cell subset, which promotes the development of allergen-specific Treg and other regulatory lymphoid cells, leading to OT [35,36]. In addition to Treg induction, anergy and T-cell depletion induce OT depending on the dose and frequency of allergen ingestion [25]. In our study, neither a decrease in the proportion of CD4^+^ cells nor an increase in the proportion of CD4^+^CD25^+^FoxP3^+^ cells was observed in the MLN and spleen of rats with OIT (Figure 6). By contrast, the increases in OVA-sIgE and sIgG_1_ levels were suppressed by daily oral administration of OVA (10 mg) for 5 days prior to immunization (Figure S1). In these rats, intravenous injection of OVA did not reduce the rectal temperature. These results were also previously observed in rats with OT to wheat gluten (10 mg) [14]. Our preliminary data showed that the proportions of CD4^+^CD25^+^FoxP3^+^ cells in the MLN and spleen of rats with OT to gluten tended to increase by approximately 10% to 40%, and the proportions of CD4^+^ cells in the spleen tended to decrease by approximately 40% compared with those in non-OT rats. These results suggest that the mechanism of desensitization in OIT may differ from the mechanism that induced OT prior to sensitization. It is necessary to obtain further molecular insights into Treg induction and shifts in T-helper cell Type 1/Type 2 balance in order to elucidate the mechanisms underlying OIT.

## 5. Conclusions

In this study, we successfully established an OIT rat model of egg allergy. In this model, OIT suppressed systemic allergic reactions associated with IgE-mediated mast cell and basophil degranulation by inducing OVA-sIgG_1_ production. Although the mechanisms underlying immunotolerance remain incompletely understood, this model provides a valuable basis for advancing our understanding of the basic mechanisms of desensitization by OIT and for optimizing therapeutic strategies for food allergies. By monitoring the dynamics of allergen-sIgG subclasses during the desensitization process, optimal treatment schedules and allergen doses can be determined to maximize therapeutic efficacy while ensuring safety.

## Data Availability

The original contributions presented in this study are included in the article/Supplementary Materials. Further inquiries can be directed to the corresponding author.

## Supplementary Materials

The following supporting information can be downloaded at: https://www.mdpi.com/article/10.3390/foods14081424/s1, Figure S1: The immunization and induction of oral tolerance to ovalbumin.

## Abbreviations

The following abbreviations are used in this manuscript:

AlphaCL	amplified luminescence proximity homogeneous assay involving crosslinking
AU	absorbance unit
BSA	bovine serum albumin
CD	cluster of differentiation
ELISA	enzyme-linked immunosorbent assay
ESI	electrospray ionization
FcεRI	high-affinity IgE receptor
Foxp3	forkhead box P3
HRP	horseradish peroxidase
IgE	immunoglobulin E
IgG	immunoglobulin G
MLN	mesenteric lymph node
OIT	oral immunotherapy
OVA	ovalbumin
PBS	phosphate-buffered saline
SE	standard error of the mean
sIgE	specific IgE
sIgG	specific IgG
TMB	3,3′,5,5′-tetramethylbenzidine
Treg	regulatory T cell
Th1/Th2	T-helper cell Type 1/Type 2
