# Supplementary figures and images for: Elucidation of Desensitization Mechanisms Induced by Oral Immunotherapy in a Rat Model of Ovalbumin Allergy
Source: Foods. 2025 Apr 21;14(8):1424. doi: 10.3390/foods14081424 (PMC12026658; doi:10.3390/foods14081424)

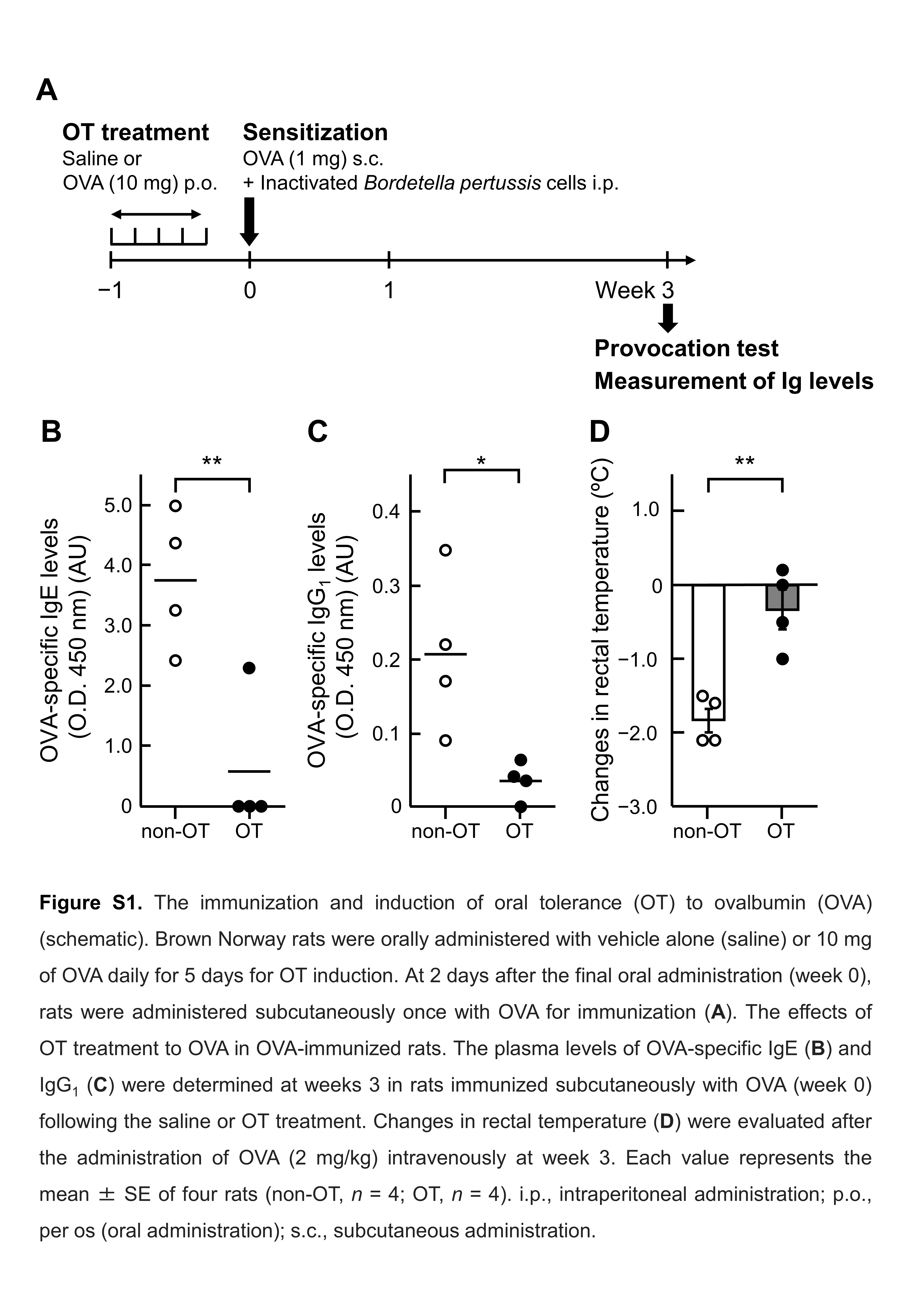

Supplement: Supplementary file 1 [file foods-14-01424-s001.zip › Supplementary Figure S1.tif]
